# Pulmonary Capillary Confinement Shapes A Motile Anuclear Neutrophil State

**DOI:** 10.21203/rs.3.rs-10269644/v3

**Published:** 2026-08-28

**Authors:** Kaori Masuhara, Yoshikazu Tsukasaki

**Affiliations:** 1 Department of Microbiology and Immunology, Louisiana State University Health Shreveport, Shreveport, Louisiana, USA

## Abstract

The nucleus is a major mechanical constraint for cells moving through confined spaces, but how organ-specific physical environments shape nuclear state and cell behavior in vivo remains unclear. Using quantitative intravital imaging with submicron stabilization of the breathing lung, we tracked neutrophil deformation, Ca^2+^ dynamics, nuclear state and motility over time. In inflamed pulmonary capillaries, neutrophil deformation was temporally coupled to transient Ca^2+^ increases, and this coupling was reduced by the mechanosensitive-channel inhibitor GsMTx4. We identified abundant neutrophil-derived cytoplasts lacking detectable nuclear signal, whose frequency was reduced by GsMTx4 and the peptidylarginine deiminase inhibitor Cl-Amidine. These cytoplasts showed greater morphological plasticity, migration speed and directional persistence than nucleated neutrophils and preferentially associated with Pseudomonas aeruginosa in vivo. Our findings identify the pulmonary capillary bed as an organ-specific mechanical environment that links cell deformation to intracellular signaling, nuclear state and immune-cell behavior.

## Introduction

The lung is optimized for gas exchange by a highly interconnected capillary network surrounding the alveoli. This network creates a thin blood–air barrier with an extensive surface area, while its narrow and highly branched lumen repeatedly constrains circulating cells. Polymorphonuclear neutrophils (PMNs) are major cells of innate immunity in the lung, but many pulmonary capillaries are similar to or smaller than the diameter of a PMN^1–3^. PMNs therefore repeatedly stop, elongate and resume movement as they pass through capillary segments, undergoing large changes in cell shape^3, 4^. This characteristic behavior contributes to the pulmonary intravascular marginated PMN pool^1, 4^ that supports rapid immune surveillance^1^, but during inflammation it may also contribute to microvascular obstruction, vascular dysfunction and tissue injury^5^.

Physical confinement within the pulmonary capillary bed may do more than restrict PMN passage; it may actively regulate intracellular signaling and functional state. Cell deformation and increased membrane tension during passage through narrow spaces can induce Ca^2+^ entry through mechanosensitive channels^6, 7^, affecting cytoskeletal remodeling and immune function^8^. In addition, PMNs that repeatedly deform within pulmonary capillaries at steady state can acquire transcriptional and functional adaptations to the lung environment through mechanosensing^9–11^. Mechanical inputs such as shear stress can also regulate PMN motility and responses associated with chromatin release^12^. Together, these findings raise the possibility that the pulmonary capillary bed is not simply a physical barrier, but an instructive physical environment that shapes PMN signaling, cell state and function.

A major factor that limits cell migration through narrow spaces is the nucleus^13^. Although the segmented PMN nucleus is highly deformable, it remains mechanically stiffer than the surrounding cytoplasm and can be a rate-limiting barrier during passage through capillaries and dense tissue spaces^14^. Changes in nuclear structure or state may therefore strongly alter the mechanical properties and migratory behavior of PMNs. In PMNs, Ca^2+^-regulated peptidylarginine deiminase (PAD) activity contributes to histone citrullination, chromatin decondensation and nuclear remodeling^15, 16^. During some non-lytic responses, PMNs can release nuclear DNA while maintaining plasma membrane integrity and transition into anuclear cytoplasts^17^. These cells have been reported to retain short-term tissue migration and bacterial uptake in vivo^17^. Experimentally generated PMN cytoplasts can also retain motility, transendothelial migration, phagocytosis and, depending on the method of generation and stimulation conditions, bactericidal activity against some bacteria^18–21^.

However, it remains unclear how naturally occurring cytoplasts emerge in relation to intracellular signaling and changes in nuclear state within an organ-specific physical environment, and how they behave after becoming anuclear. The pulmonary capillary bed, which repeatedly deforms PMNs, may provide a physiological mechanical environment that links cell deformation to Ca^2+^ dynamics and changes in nuclear state, while also shaping the behavior of cells after transition to an anuclear state. Directly tracking these events in cells within the living lung has been difficult because of the large tissue motion artifacts including displacement and deformation caused by respiration. Integrated analysis of cell deformation, intracellular signaling, nuclear state and migratory behavior therefore requires intravital imaging that can stabilize the breathing lung and quantify individual cells over time.

Here, we used Computer-vision Stabilized intravital imaging (CoVSTii)^3^, a motion-correction intravital imaging approach that stabilizes images of the breathing lung with submicron precision, to track individual PMNs moving through pulmonary capillaries. This platform allowed us to quantify cell deformation, Ca^2+^ activity, nuclear state and migratory behavior along the same spatiotemporal axis. In the inflamed lung, we identified GsMTx4-sensitive Ca^2+^ signals that were temporally coupled to PMN deformation and found that PMN-derived cytoplasts lacking detectable nuclear signal were frequent in the pulmonary microvasculature. Their appearance was associated with GsMTx4-sensitive signaling and PAD-related nuclear remodeling. We further found that these cytoplasts showed distinct morphological plasticity, motility and bacterial association compared with nucleated PMNs. These findings position the pulmonary capillary bed not simply as a structure that passively traps circulating cells, but as an organ-specific mechanical environment that links cell deformation, intracellular signaling, nuclear state and dynamic cell behavior in space and time.

## Results

### PMN deformation in pulmonary capillaries is temporally coupled to GsMTx4-sensitive Ca^2+^ signaling

Pulmonary capillaries form a narrow vascular network with dimensions comparable to the diameter of PMNs, and their passage through this network requires substantial cell deformation. We reasoned that this mechanical confinement within capillaries might be associated with PMN Ca^2+^ signaling. To examine PMN Ca^2+^ dynamics in the capillaries of inflamed lungs, we performed two-photon intravital Ca^2+^ imaging in mechanically ventilated mice. The living lung was imaged through a suction-stabilized thoracic window^22^, and CoVSTii motion correction was performed using CD31-labeled pulmonary vascular structures as a structural reference^3^. We used Catchup-Salsa6f reporter^23, 24^ mice expressing GCaMP6f–tdTomato specifically in PMNs and quantified ratiometric Ca^2+^ dynamics in vivo based on the GCaMP6f/tdTomato ratio.

**Figure 1:**
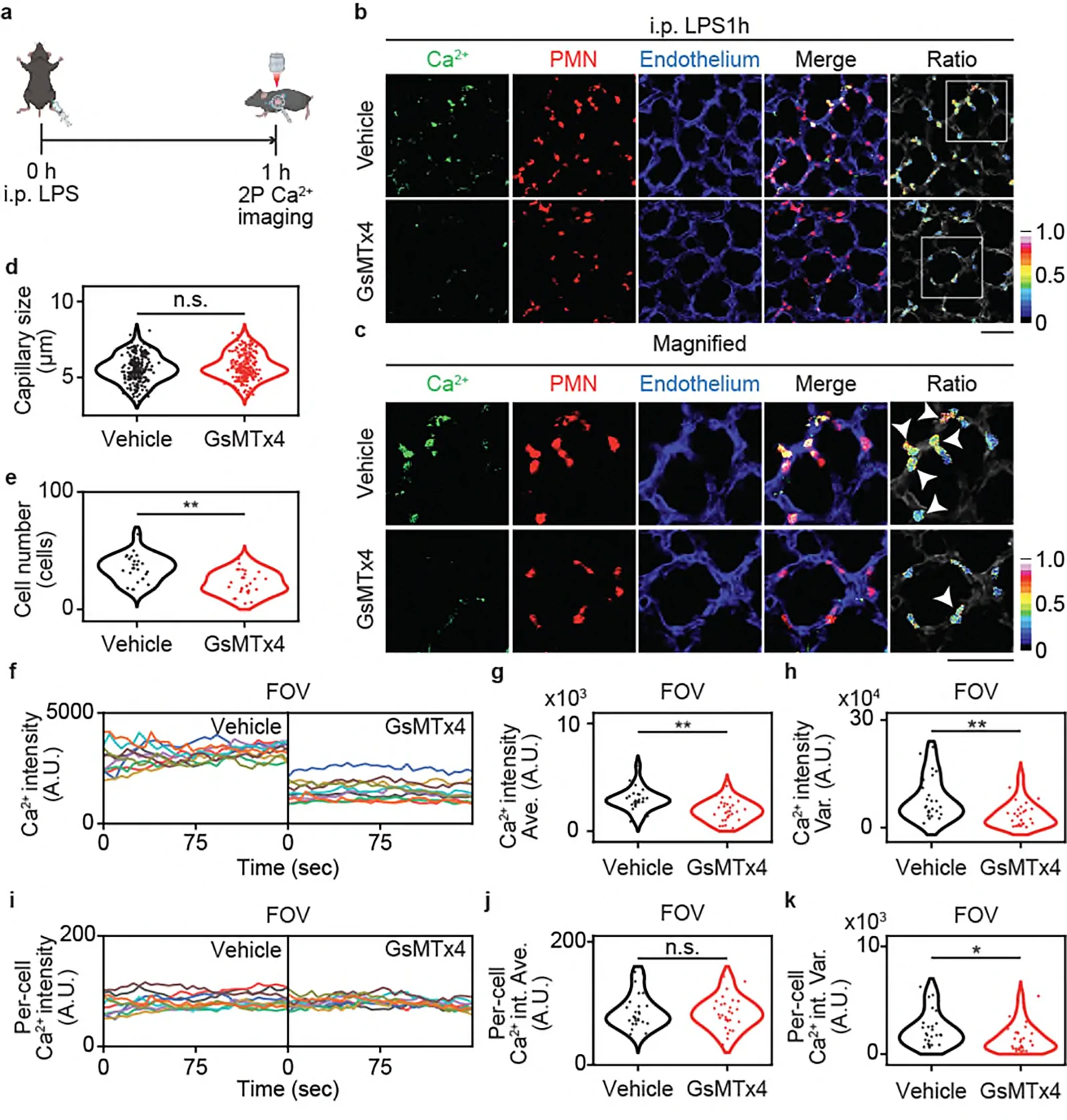
PMN Ca^2+^ dynamics in pulmonary capillaries are GsMTx4-sensitive. **a**, Experimental overview. LPS was administered intraperitoneally, and two-photon intravital Ca^2+^ imaging was performed 1 h later. **b**, Representative two-photon and ratiometric Ca^2+^ images of PMNs in the pulmonary microvasculature of Vehicle- or GsMTx4-treated mice. Ca^2+^ (GCaMP6f, green), PMNs (tdTomato, red), pulmonary vascular endothelium (blue), Merge and GCaMP6f/tdTomato ratio are shown. In the ratio image, white indicates pulmonary vascular structures. Scale bar, 50 μm. **c**, Enlarged images of the white-boxed region in **b**. Arrowheads indicate PMNs with increased Ca^2+^ signal. Scale bar, 50 μm. **d**, Quantification of pulmonary capillary diameter in Vehicle- and GsMTx4-treated groups. **e**, Number of PMNs per field of view (FOV). **f**, Representative time courses of FOV-level Ca^2+^ intensity in the Vehicle (left) and GsMTx4 (right) groups 1 h after LPS administration. **g**,**h**, Quantification of mean Ca^2+^ signal intensity (**g**) and temporal variance of the Ca^2+^ signal (**h**) in each FOV. **i**, Representative time course of Ca^2+^ signal intensity normalized to cell number. **j**,**k**, Quantification of mean Ca^2+^ signal intensity (**j**) and temporal variance (**k**) after normalization to cell number. d, Three mice; Vehicle, 26 FOVs and 207 vessels; GsMTx4, 29 FOVs and 217 vessels. e,g,h,j,k, Three mice; Vehicle, 26 FOVs; GsMTx4, 29 FOVs. Statistical significance was assessed using unpaired two-sided Studenťs t-tests. *P < 0.05, **P < 0.01; n.s., not significant.

One hour after intraperitoneal LPS administration, PMNs accumulated within the pulmonary microvasculature and individual cells showed repetitive Ca^2+^ fluctuations (Fig. 1a–c and **supplementary video 1**). To test whether mechanosensitive signaling contributes to Ca^2+^ responses during pulmonary capillary passage, we administered the mechanosensitive ion-channel inhibitor peptide GsMTx4^25^. GsMTx4 did not significantly affect pulmonary capillary diameter (Vehicle, 5.59 ± 0.06 μm; GsMTx4, 5.74 ± 0.06 μm; mean ± s.e.; P=0.09; Fig. 1d) but reduced the number of PMNs per field of view to approximately 64% of the Vehicle value (Vehicle, 35.2 ± 2.2 cells; GsMTx4, 22.5 ± 1.8 cells; mean ± s.e.; Fig. 1e). At the field-of-view level, both mean Ca^2+^ signal intensity and its temporal variance were reduced by GsMTx4 treatment to 0.64-fold and 0.46-fold of Vehicle, respectively (Intensity average: Vehicle, 2,953.8 ± 204.0 A.U.; GsMTx4, 1,887.5 ± 176.2 A.U.; Intensity variance: Vehicle, 79,013.9 ± 11,464.3 A.U.; GsMTx4, 35,960.8 ± 6,495.6 A.U.; mean ± s.e.; Fig. 1f–h and **supplementary video 1**). Because the reduced PMN number could contribute to this decrease, we next analyzed Ca^2+^ activity normalized to cell number (Fig. 1i–k). Mean Ca^2+^ signal intensity per cell was lower in the GsMTx4 group but did not differ significantly (Vehicle, 86.9 ± 5.4 A.U.; GsMTx4, 84.3 ± 4.8 A.U.; mean ± s.e.; P = 0.72; Fig. 1i,j). In contrast, temporal variance of the per-cell Ca^2+^ signal was significantly reduced to 0.64-fold of the Vehicle value (Vehicle, 2,291.7 ± 310.2 A.U.; GsMTx4, 1,469.5 ± 238.4 A.U.; mean ± s.e.; Fig. 1k). These results suggest that GsMTx4 does not uniformly lower PMN Ca^2+^ levels but preferentially reduces dynamic Ca^2+^ fluctuations in the pulmonary microvasculature.

**Figure 2:**
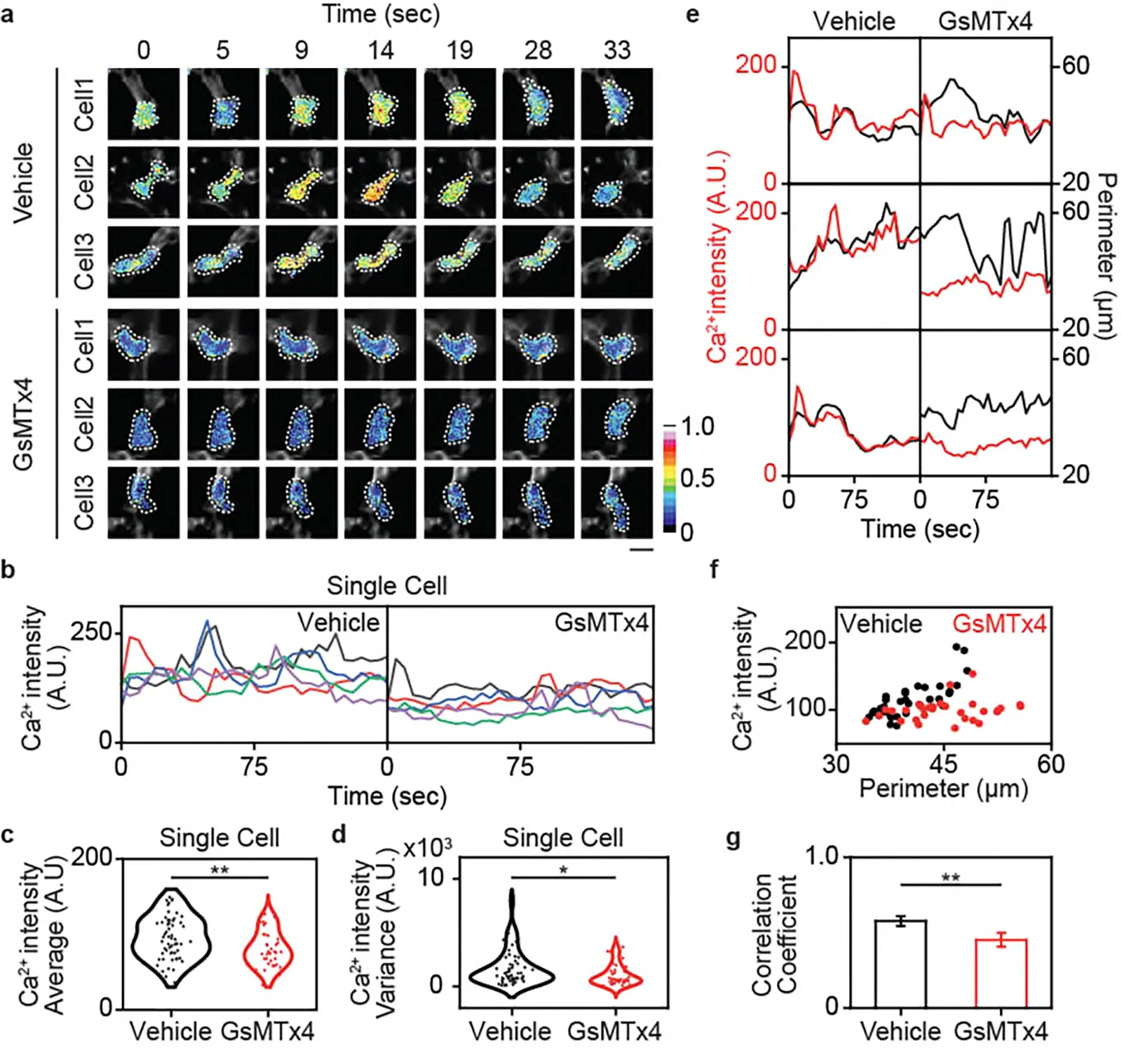
PMN deformation is temporally coupled to a GsMTx4-sensitive Ca^2+^ response. **a**, Representative single-cell ratiometric Ca^2+^ time-series images of PMNs moving through pulmonary capillaries in Vehicle- or GsMTx4-treated mice. White dashed lines indicate cell outlines. White indicates pulmonary vascular structures. Scale bar, 10 μm. **b**, Representative time courses of single-cell Ca^2+^ intensity in the Vehicle (left) and GsMTx4 (right) groups 1 h after LPS administration. **c**,**d**, Quantification of mean Ca^2+^ signal intensity (**c**) and temporal variance of the Ca^2+^ signal (**d**) in individual PMNs. **e**, Representative time courses of Ca^2+^ intensity (red) and cell perimeter (black) in a single PMN from the Vehicle (left) and GsMTx4 (right) groups. **f**, Representative scatter plots showing the relationship between Ca^2+^ intensity and cell perimeter. **g**, Comparison of correlation coefficients between Ca^2+^ intensity and cell perimeter. c,d,g, Three mice; Vehicle, 26 FOVs and 57 cells; GsMTx4, 29 FOVs and 35 cells. Statistical significance was assessed using unpaired two-sided Studenťs t-tests. *P < 0.05, **P < 0.01.

We next examined at the single-cell level whether these GsMTx4-sensitive Ca^2+^ fluctuations were temporally associated with PMN deformation during pulmonary capillary passage. Tracking individual PMNs moving through pulmonary capillaries revealed frequent transient increases in intracellular Ca^2+^ as cells entered narrow or branched capillary segments and underwent marked changes in shape, including elongation (Fig. 2a,b and **supplementary video 2**). After GsMTx4 treatment, these deformation-associated Ca^2+^ increases were reduced (Fig. 2a,b and **supplementary video 2**). Single-cell tracking analysis also showed significant reductions in both mean Ca^2+^ signal intensity and temporal variance after GsMTx4 treatment (Fig. 2c,d). To quantify the temporal relationship between cell deformation and Ca^2+^ activity, we used cell perimeter as a measure of dynamic shape change. Under Vehicle conditions, changes in Ca^2+^ fluorescence intensity tracked with changes in cell perimeter: Ca^2+^ activity tended to increase as cells elongated and to decrease as they returned to a more compact shape (Fig. 2e). This temporal coupling was weaker after GsMTx4 treatment (Fig. 2e). Consistent with this observation, Ca^2+^ fluorescence intensity was positively correlated with cell perimeter, and the correlation coefficient was significantly reduced after GsMTx4 treatment (Vehicle, 0.58 ± 0.03; GsMTx4, 0.45 ± 0.05; mean ± s.e.; Fig. 2f,g).

Together, these results show that dynamic PMN deformation within capillaries of the inflamed lung is temporally coupled to GsMTx4-sensitive Ca^2+^ responses.

### GsMTx4-sensitive signaling and PAD activity are associated with nuclear remodeling and PMN_cyt_ appearance

We next asked whether the GsMTx4-sensitive signaling observed during capillary passage was associated with PMN nuclear state. To visualize PMN nuclei in vivo, we intravenously administered the cell-permeable nuclear dye SYTO40 to Catchup reporter mice three hours after LPS administration and performed two-photon intravital imaging.

**Figure 3:**
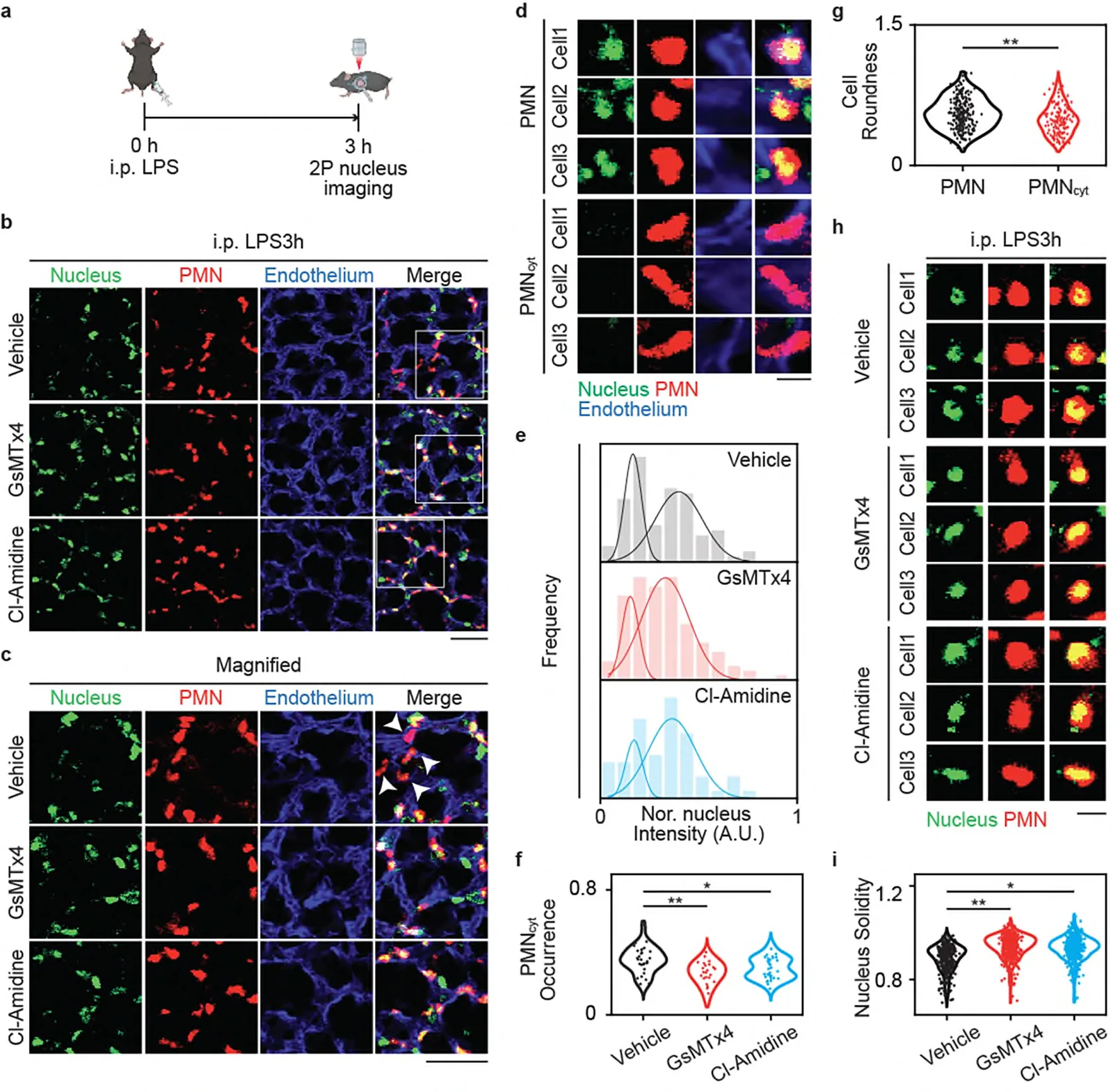
GsMTx4-sensitive signaling and PAD activity are associated with PMN nuclear remodeling and PMN_cyt_ appearance. **a**, Experimental overview. LPS was administered intraperitoneally, and two-photon intravital nuclear imaging was performed 3 h later. **b**, Representative two-photon images showing nuclei (SYTO40, green), PMNs (red) and pulmonary microvessels (blue) 3 h after LPS in Vehicle-, GsMTx4- and Cl-Amidine-treated mice. Scale bar, 50 μm. **c**, Enlarged images of the white-boxed region in **b**. Arrowheads indicate PMN-derived cells lacking detectable nuclear signal. Scale bar, 50 μm. **d**, Representative single-cell images of nucleated PMNs and PMN_cyt_s. Scale bar, 10 μm. **e**, Distribution of normalized nuclear fluorescence intensity in Vehicle-, GsMTx4- and Cl-Amidine-treated groups. **f**, Quantification of PMN_cyt_ frequency in each treatment group. **g**, Quantification of cell roundness in nucleated PMNs and PMN_cyt_s. **h**, Representative nuclear morphology of nucleated PMNs in Vehicle-, GsMTx4- and Cl-Amidine-treated groups. Scale bar, 10 μm. **i**, Quantification of nuclear solidity in each treatment group. f,i, Three mice; Vehicle, 31 FOVs and 419 cells; GsMTx4, 25 FOVs and 484 cells; Cl-Amidine, 34 FOVs and 552 cells. g, Three mice, 10 FOVs; PMN, 243 cells; PMN_cyt_, 128 cells. Statistical significance was assessed using one-way ANOVA followed by Tukey's multiple-comparisons test (f,i) or unpaired two-sided Studenťs t-test (g). *P < 0.05, **P < 0.01; n.s., not significant.

Within the microvasculature of inflamed lungs, we frequently observed PMN-derived cells lacking detectable nuclear fluorescence in addition to PMNs with clearly segmented nuclei (Fig. 3a–d and **supplementary videos 3 and 4**). Single-cell analysis of SYTO40 fluorescence intensity distinguished cells with clear nuclear signals from a population with low or undetectable nuclear signals (Fig. 3e). The latter retained a continuous cell body rather than dispersed fluorescent fragments, and their morphology was consistent with previously described PMN-derived cytoplasts^17^. We therefore operationally defined these cells as PMN cytoplasts (PMN_cyt_s). Three hours after LPS administration, PMN_cyt_s accounted for 35.2 ± 1.5% of the PMN population observed in the pulmonary microvasculature (Fig. 3f). PAD-dependent chromatin remodeling is known to contribute to structural changes in the PMN nucleus^15, 16^. To examine the relationship of this PMN_cyt_ state with GsMTx4-sensitive signaling and PAD activity, we treated mice with GsMTx4 or the PAD inhibitor Cl-Amidine^26^. GsMTx4 reduced the frequency of PMN_cyt_s to 26.8 ± 1.5%, whereas Cl-Amidine reduced it to 29.8 ± 1.2% (mean ± s.e.; Fig. 3f). These results suggest that GsMTx4-sensitive signaling and Cl-Amidine-sensitive PAD activity are associated with the appearance or maintenance of the PMN_cyt_ state in the inflamed lung. In addition to the loss of detectable nuclear signal, PMN_cyt_s showed whole-cell morphological features distinct from those of nucleated PMNs. Whereas nucleated PMNs were mainly round or compact, PMN_cyt_s more frequently showed an elongated morphology (Fig. 3b–d). Consistent with this observation, roundness was significantly lower in PMN_cyt_s than in nucleated PMNs (PMN, 0.56 ± 0.01; PMN_cyt_s, 0.49 ± 0.01; mean ± s.e.; Fig. 3g). Thus, PMN_cyt_s were characterized by both the absence of detectable nuclear signal and a more elongated cell shape.

We further examined whether GsMTx4 and Cl-Amidine also affected the morphology of nuclei that remained in nucleated PMNs. In Vehicle-treated mice, PMNs often showed irregular, highly segmented nuclei, whereas nuclei were more compact after GsMTx4 or Cl-Amidine treatment (Fig. 3h). Consistent with this observation, nuclear solidity increased significantly under both conditions (Vehicle, 0.894 ± 0.003; GsMTx4, 0.943 ± 0.002; Cl-Amidine, 0.936 ± 0.002; mean ± s.e.; Fig. 3i).

Together, these results suggest that both GsMTx4-sensitive signaling and PAD activity are associated with structural remodeling of the PMN nucleus and with the PMN_cyt_ state.

### PMN cytoplasts show increased temporal morphological plasticity and persistent motility

The nucleus can be a major mechanical constraint for PMNs moving through narrow pulmonary capillaries and may influence their shape changes and migratory behavior. We therefore asked whether the elongated morphology observed in PMN_cyt_s reflected a stable morphological feature or increased dynamic remodeling. Individual cells were tracked over time using cytoplasmic tdTomato fluorescence in PMNs. Cell segmentation masks were generated at each time point, and cell perimeter and centroid position were quantified from the image sequences.

**Figure 4:**
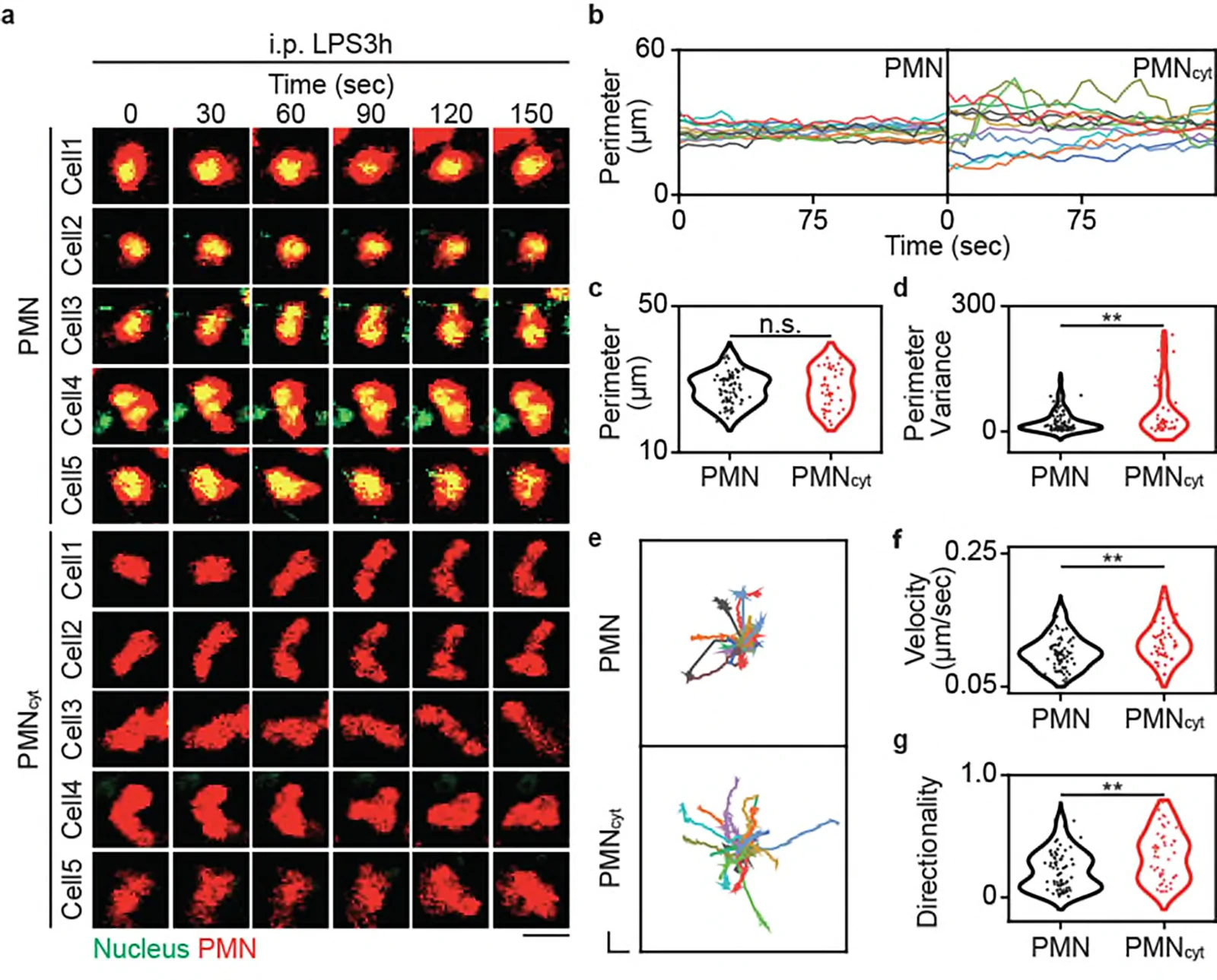
PMN-derived cytoplasts show high morphological plasticity and persistent motility. **a**, Representative time-lapse images of nucleated PMNs and PMN_cyt_s 3 h after LPS administration. Nuclei (green) and PMNs (red) are shown. Scale bar, 10 μm. **b**, Representative time courses of cell perimeter in a nucleated PMN (left) and a PMN_cyt_ (right). **c**, Mean cell perimeter of nucleated PMNs and PMN_cyt_s. **d**, Temporal variation in cell perimeter quantified as variance. **e**, Representative migration tracks of nucleated PMNs and PMN_cyt_s. **f**, Quantification of migration speed. **g**, Quantification of directional persistence, defined as the ratio of net displacement to total path length. c,d,f,g, Three mice and 9 FOVs; PMN, 74 cells; PMN_cyt_, 42 cells (c,d) or 48 cells (f,g). Statistical significance was assessed using unpaired two-sided Studenťs t-tests. **P < 0.01; n.s., not significant.

Nucleated PMNs transiently deformed while passing through capillary branch points but otherwise maintained a relatively stable morphology during the observation period (Fig. 4a,b and **supplementary video 5**). In contrast, PMN_cyt_s repeatedly elongated and contracted, with rapid changes in cell contour accompanied by extension and retraction of protrusion-like structures (Fig. 4a,b and **supplementary video 5**). Mean cell perimeter did not differ significantly between nucleated PMNs and PMN_cyt_s (PMN, 27.4 ± 0.51 μm; PMN_cyt_s, 27.6 ± 0.85 μm; mean ± s.e.; P = 0.78; Fig. 4c). In contrast, temporal variance of cell perimeter was approximately two-fold higher in PMN_cyt_s (PMN, 22.5 ± 2.8; PMN_cyt_s, 44.9 ± 8.8; Fig. 4d). Thus, the elongated morphology observed in PMN_cyt_s could not be explained by a fixed shape difference alone but instead reflected high temporal morphological plasticity with continuous remodeling of the cell boundary.

We next examined whether this increased morphological plasticity was accompanied by altered migratory behavior by tracking cell centroids over time. PMN_cyt_s moved faster than nucleated PMNs and also showed greater directional persistence, defined as the ratio of net displacement to total path length (Fig. 4e–g and **supplementary video 4**). Nucleated PMNs frequently paused or changed direction locally during short-range movement, whereas PMN_cyt_s tended to move more persistently through consecutive capillary segments. PMN_cyt_ migration speed was approximately 1.2-fold higher than that of nucleated PMNs (PMN, 0.10 ± 0.003 μm s^−1^; PMN_cyt_s, 0.12 ± 0.004 μm s^−1^; mean ± s.e.; Fig. 4f), and directional persistence was increased by approximately 1.5-fold (PMN, 0.23 ± 0.02; PMN_cyt_s, 0.34 ± 0.03; mean ± s.e.; Fig. 4g).

These results show that PMN_cyt_s are not simply static anuclear cells but represent a dynamically distinct PMN-derived cell state with high temporal morphological plasticity and persistent motility in the pulmonary microvasculature.

### PMN cytoplasts preferentially associate with bacteria in vivo

**Figure 5:**
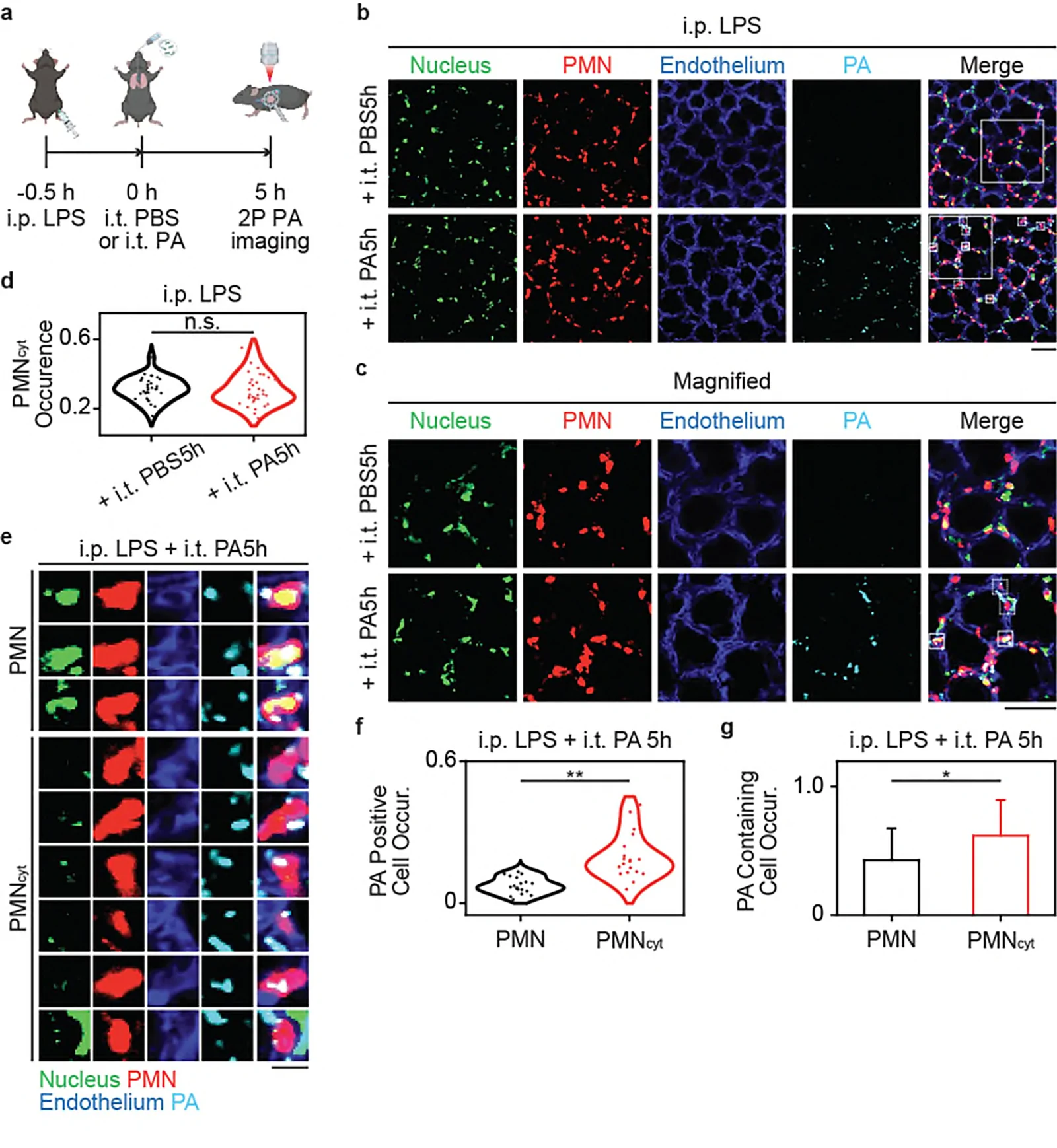
PMN-derived cytoplasts preferentially associate with bacteria during early pulmonary infection. a, Experimental overview. LPS was administered intraperitoneally, followed 30 min later by intratracheal administration of PBS or GFP-expressing *Pseudomonas aeruginosa* (GFP-PA), and two-photon intravital imaging was performed 5 h later. **b**, Representative intravital images showing nuclei (green), PMNs (red), pulmonary microvessels (blue) and PA (cyan) 5 h after PBS or GFP-PA administration. Large white boxes indicate regions enlarged in **c**. Small white boxes indicate regions enlarged in **e**. Solid small white boxes indicate nucleated PMNs associated with GFP-PA, and dashed small white boxes indicate PMN_cyt_s associated with GFP-PA. Scale bar, 50 μm. **c**, Enlarged image of the large white-boxed region in b. Scale bar, 50 μm. **d**, Quantification of PMN_cyt_ frequency after PBS or GFP-PA administration. **e**, Representative enlarged images of nucleated PMNs and PMN_cyt_s associated with GFP-PA in the small white-boxed regions shown in **b** and **c**. Scale bar, 10 μm. **f**, Proportion of nucleated PMNs and PMN_cyt_s associated with GFP-PA. **g**, Proportion of nucleated PMNs and PMN_cyt_s containing GFP-PA signal within the segmented cell boundary. d, i.t. PBS, three mice and 32 FOVs; i.t. PA, four mice and 35 FOVs. f,g, Four mice and 17 FOVs. Statistical significance was assessed using unpaired two-sided Studenťs t-tests. *P < 0.05, **P < 0.01; n.s., not significant.

We next asked whether the characteristic morphological plasticity and migratory behavior of PMN_cyt_s were accompanied by altered interactions with bacteria. Catchup reporter mice were treated with LPS to induce lung inflammation, followed by intratracheal administration of GFP-expressing *Pseudomonas aeruginosa*. Two-photon intravital imaging was performed five hours after bacterial administration, and PMN–bacteria interactions in the pulmonary microvasculature were quantified (Fig. 5a–c). PMN_cyt_ frequency did not differ significantly between PBS-treated controls and *P. aeruginosa*-exposed mice (PBS, 31.3 ± 1.2%; *P. aeruginosa*, 30.8 ± 1.6%; mean ± s.e.; P = 0.81; Fig. 5d). Thus, within the time window analyzed, bacterial exposure itself did not rapidly increase the PMN_cyt_ population. We next defined bacterial association as the presence of GFP–*P. aeruginosa* signal within or immediately adjacent to a segmented PMN mask and quantified its frequency. Compared with nucleated PMNs in the same pulmonary microenvironment, PMN_cyt_s associated with GFP–*P. aeruginosa* 2.7-fold more frequently (PMN, 7.5 ± 0.9%; PMN_cyt_s, 20.5 ± 2.3%; mean ± s.e.; Fig. 5e,f). We then applied a more stringent criterion requiring bacterial GFP signal to be located within the segmented cell boundary. The proportion of cells containing bacterial signal within the cell boundary was also higher in PMN_cyt_s than in nucleated PMNs, increasing by approximately 1.4-fold (PMN, 42.8 ± 6.0%; PMN_cyt_s, 61.8 ± 6.8%; Fig. 5e,g). Thus, PMN_cyt_s not only associated with bacteria more frequently but also more often contained bacterial fluorescence within their cell boundary.

Together, these results show that PMN_cyt_s represent a PMN-derived cell state with behavior distinct from nucleated PMNs in the inflamed lung and with a higher frequency of bacterial association.

## Discussion

**Figure 6:**
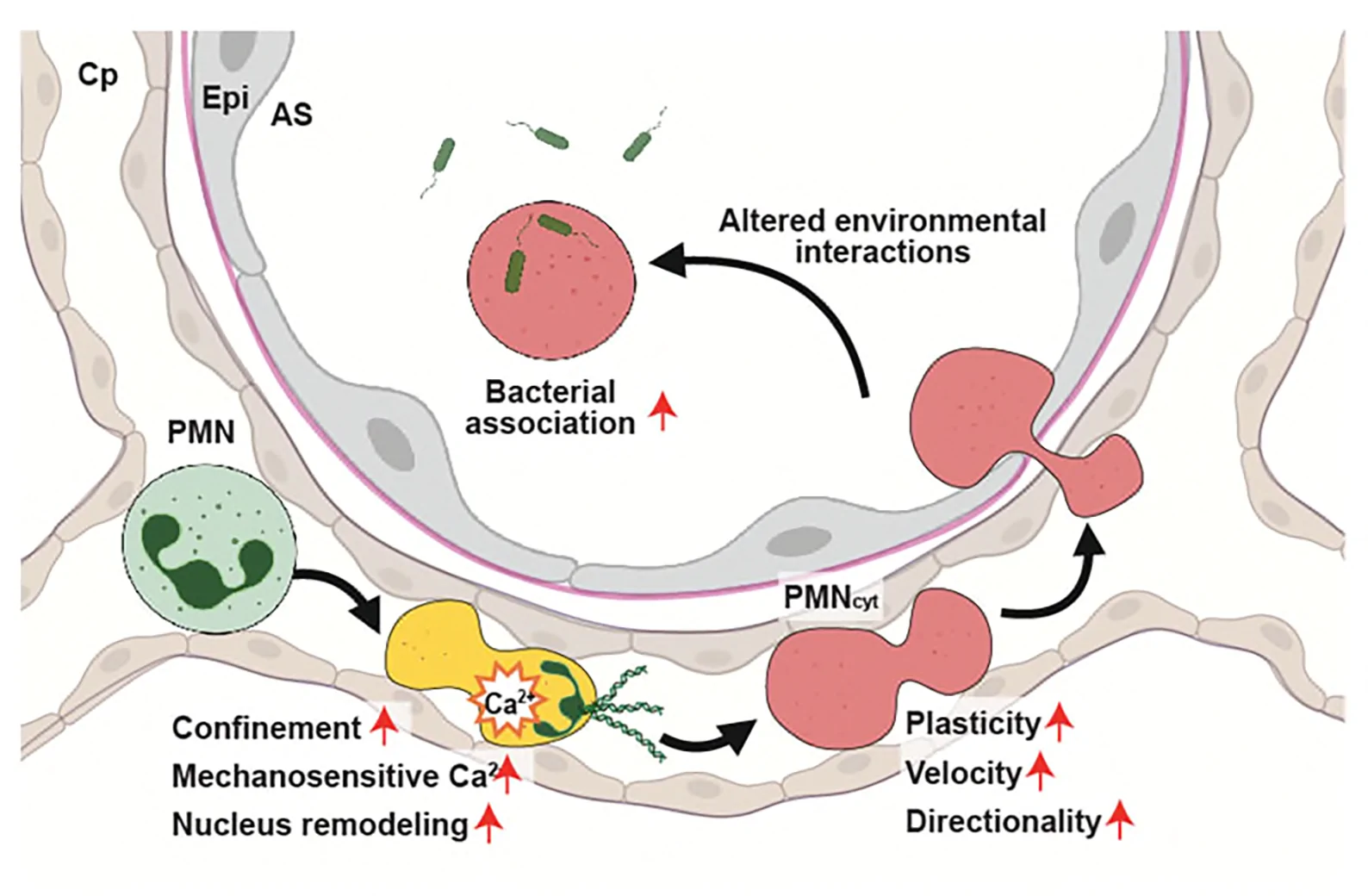
Model of PMN_cyt_ appearance and bacterial interactions associated with pulmonary capillary confinement. During recruitment to the inflamed lung, PMNs repeatedly deform while passing through narrow pulmonary capillaries. This physical confinement is associated with GsMTx4-sensitive mechanosensitive Ca^2+^ responses, nuclear remodeling and the appearance of anuclear PMN-derived cytoplasts (PMN_cyt_s). PMN_cyt_s show high morphological plasticity, migration speed and directional persistence, together with altered interactions with the pulmonary microenvironment. These properties may increase the probability of encountering and associating with bacteria within the spatially constrained inflamed lung. CP, Capillary; Epi, Epithelium; AS, Alveolar space.

This study visualized and quantified PMN intracellular signaling, nuclear state and single-cell dynamics that have been difficult to analyze in an integrated manner in the breathing lung and defined an anuclear PMN state characterized by high morphological plasticity, persistent motility and distinct environmental interactions. By tracking individual PMNs under respiratory motion, we found that transient Ca^2+^ increases accompanied cell deformation during capillary passage in the inflamed lung and that this temporal coupling was reduced by GsMTx4 treatment. Intravital visualization of nuclei revealed frequent PMN-derived cytoplasts lacking detectable nuclear signal in the pulmonary microvasculature, and both their frequency and nuclear morphology in nucleated PMNs were altered by GsMTx4 and Cl-Amidine. PMN_cyt_s also showed greater morphological plasticity, migration speed and directional persistence than nucleated PMNs and preferentially associated with *P. aeruginosa* in vivo. Together, these results support a model in which the pulmonary capillary bed acts as an organ-specific mechanical environment that links repetitive cell deformation to intracellular signaling, nuclear state and dynamic cell behavior (Fig. 6). A defining feature of the pulmonary microcirculation is that PMNs repeatedly deform as they move through narrow and highly branched capillary segments^3, 4^. In the steady-state lung, deformation of PMNs during capillary passage has been linked to spontaneous Ca^2+^ activity, and repeated passage through constrictions has been reported to induce mechanosensitive responses distinct from those triggered by a single constriction^11^. Here, we show that cell deformation and Ca^2+^ activity are also temporally coupled at the single-cell level in the inflamed lung. Together, these findings suggest that the pulmonary capillary network does more than passively retain circulating PMNs; it may act as a dynamic mechanical niche that converts the history of repeated cell deformation into intracellular signals.

In single-cell tracking analyses, GsMTx4 reduced both Ca^2+^ signal intensity and temporal fluctuations and weakened the correlation between Ca^2+^ activity and cell deformation, supporting the involvement of a GsMTx4-sensitive mechanosensitive pathway in this temporal coupling. However, GsMTx4 can act on multiple mechanosensitive ion channels^25^, and our study does not identify the responsible channel. In addition, Ca^2+^ pathways downstream of chemokine receptors and pattern-recognition receptors may operate in parallel in the inflamed lung^27, 28^. The Ca^2+^ dynamics observed here are therefore likely to reflect the integration of multiple signals in vivo, including mechanosensitive input. Even within this complex signaling environment, our study directly shows that PMN deformation during pulmonary capillary passage is temporally coupled to Ca^2+^ dynamics at the single-cell level.

The effects of GsMTx4 and Cl-Amidine on both PMN_cyt_ frequency and nuclear morphology in nucleated PMNs indicate that GsMTx4-sensitive signaling and PAD activity are associated with nuclear remodeling and the PMN_cyt_ state in the inflamed lung. PAD activity contributes to Ca^2+^-dependent histone citrullination, chromatin decondensation and nuclear remodeling, and the more compact nuclei observed after Cl-Amidine treatment are consistent with reduced drive toward nuclear decondensation^15, 16^. The similar changes observed after GsMTx4 treatment are also consistent with a possible functional link between GsMTx4-sensitive Ca^2+^ signaling and PAD-related nuclear remodeling. However, we did not continuously visualize the complete sequence from deformation-associated Ca^2+^ elevation to changes in nuclear structure and loss of nuclear DNA in the same cell. Thus, the pharmacological findings support a functional association among these events but do not establish a complete causal pathway.

The finding that PMN_cyt_s accounted for approximately 30–40% of the PMN population in inflamed lungs indicates that the anuclear state is not a rare terminal event but a frequently observed PMN state in the pulmonary microvasculature. Anuclear PMNs generated after neutrophil extracellular trap (NET) release have also been reported to continue moving and taking up bacteria in vivo^17^. Experimentally generated PMN cytoplasts can retain Ca^2+^ mobilization, actin remodeling, spontaneous or chemotactic movement, phagocytosis and, under some conditions, bactericidal activity against certain bacteria^18–21^. However, directional migration and function of cytoplasts have not been consistent across previous studies and vary with the method of generation and tissue environment. The high morphological plasticity, migration speed and directional persistence of PMN_cyt_s observed here may therefore represent a distinct dynamic state that emerges within the pulmonary capillary environment. Reduced mechanical constraint due to the absence of the relatively stiff nucleus may facilitate shape adaptation and persistent movement through the highly branched capillary network^13, 14^. However, PMN_cyt_ behavior is unlikely to be determined by nuclear loss alone and may instead emerge from the combined effects of intracellular signaling, cytoskeletal organization, adhesion, membrane mechanics, local blood flow and the structural environment of pulmonary capillaries^29, 30^.

The preferential association of PMN_cyt_s with *P. aeruginosa*, together with their more frequent retention of bacterial signal within the cell boundary, indicates that this mechanically and dynamically distinct cell state is linked to altered local host–pathogen interactions. High morphological adaptability and persistent motility may increase opportunities to approach and associate with bacteria within the complex pulmonary microenvironment^7, 31^. However, because bacterial viability and killing were not assessed in this study, increased bacterial association cannot be interpreted as enhanced antimicrobial activity or bacterial clearance. What our study directly shows is that a quantitatively defined anuclear PMN state exhibits environmental interactions in the living lung that differ from those of nucleated PMNs. The subsequent fate and function of this cell state remain unclear. It is not known how long PMN_cyt_s survive in the living lung, how they are removed, or whether they localize to different pulmonary compartments. It also remains unknown to what extent they retain phagocytic or bactericidal function after bacterial association and how they contribute to host defense, inflammatory control or tissue injury.

This study visualized and quantified PMN intracellular dynamics that have been difficult to analyze directly in the breathing lung, linking cell deformation within pulmonary capillaries to Ca^2+^ signaling, nuclear remodeling and dynamic behavior, and identifying an anuclear PMN state characterized by high morphological plasticity, persistent motility and distinct environmental interactions. These findings suggest that the pulmonary capillary bed is not simply a route of circulation or a physical filter, but an organ-specific mechanical environment that can shape immune-cell state and function.

## Online methods

### Mice

Mice were housed under specific-pathogen-free conditions in the Research Resources Laboratory at the University of Illinois Chicago (UIC) and the Animal Resources facility at Louisiana State University Health Shreveport (LSU Health Shreveport). All animal experiments were performed under protocols approved by the Institutional Animal Care and Use Committees of UIC and LSU Health Shreveport (UIC protocol 24–039; LSU protocol P-26-029). Male and female mice 10–16 weeks of age were used. Catchup mice were provided by Dr. Gunzer at the University Duisburg–Essen under a material transfer agreement^23^. PMN-specific Salsa6f reporter mice were generated by crossing Catchup mice with Salsa6f mice (strain 031968, Jackson Laboratory)^24^.

### Endotoxaemia and bacterial infection

To induce acute lung inflammation, lipopolysaccharide (LPS) from *Escherichia coli* O111 (L2630, Sigma-Aldrich) was dissolved in PBS and administered intraperitoneally at 10 mg kg^−1^. Ca^2+^ imaging was performed 1 h after LPS administration, and nuclear-state and PMN_cyt_ analyses were performed 3 h after LPS administration. GFP-expressing *Pseudomonas aeruginosa* PAO1 was provided by Dr. Reddy at UIC and cultured overnight at 37 °C in LB medium containing ampicillin (100 g ml^−1^; A-301-5, GoldBio)^7^. Bacterial concentration was determined by serial dilution, plating on LB–ampicillin agar and colony-forming unit (CFU) counting. For bacterial infection, 1 × 10^8^ CFU in 40 μl PBS was administered intratracheally 30 min after LPS using a micro-aerosol sprayer (BJ-PW-M, Bio Jane BioTech). Lung intravital imaging of bacteria was performed 5 h after bacterial administration.

### Pharmacological inhibition

GsMTx4 (AB141871, Abcam) and Cl-Amidine (506282, Sigma-Aldrich) were used to inhibit mechanosensitive channel activity^25^ and PAD activity^26^, respectively. GsMTx4 and Cl-Amidine were dissolved in PBS, and PBS was used as the vehicle control. GsMTx4 was administered intraperitoneally at 270 g kg^−1^ at 2 h and 1 h before LPS administration. Cl-Amidine was administered intraperitoneally at 50 mg kg^−1^ 30 min before LPS administration.

### Lung intravital imaging

Surgical preparation of the mouse lung and two-photon intravital imaging were performed as described previously^3^. Catchup-Salsa6f mice were used for Ca^2+^ imaging, and Catchup mice were used for nuclear and bacterial imaging. To visualize the pulmonary microvasculature, SeTau647 (K9-4149, SETA Biomedicals)-labeled anti-CD31 antibody (102402, clone 390, BioLegend; 10 μg per mouse) was administered intravenously through the retro-orbital sinus. To visualize nuclei, SYTO40 (0.167 mM; 5 μl per mouse) (S11351, Thermo Fisher Scientific) was administered through the same route. Time-lapse images were acquired at 30–40 frames s^−1^ using a Leica Stellaris 8 DIVE (25×, NA 1.00), Bruker Ultima In Vivo (20×, NA 1.0) or Olympus FVMPE-RS (25×, NA 1.05) multiphoton microscope. For Ca^2+^ imaging, GCaMP, tdTomato and SeTau647 signals were simultaneously acquired in separate detection channels using 940-nm excitation. For nuclear and bacterial imaging, SYTO40, GFP, tdTomato and SeTau647 signals were simultaneously acquired in separate detection channels using 860-nm excitation.

### Image analysis

Time-lapse images were analyzed using a custom LabVIEW program (National Instruments) and ImageJ. Respiratory motion artifacts were corrected with CoVSTii^3^ using the pulmonary structural signal from SeTau647-labeled anti-CD31 antibody as the reference channel. Ratiometric Ca^2+^ signals were calculated as the GCaMP/tdTomato ratio. Individual PMNs were tracked over time, and Ca^2+^ intensity and cell perimeter were quantified. Correlation coefficients between Ca^2+^ intensity and cell perimeter were calculated from paired time-series measurements for each cell, and Fisher z-transformed values were used for group comparisons. PMNs and PMN_cyt_s were classified using a threshold defined by the intersection of two Gaussian components obtained by fitting the distribution of SYTO40 nuclear fluorescence intensity with a multi-Gaussian model. Cell and nuclear morphology were quantified from segmentation masks. Cell roundness was calculated as minor-axis length divided by major-axis length, and nuclear solidity as nuclear area divided by convex-hull area. For cell-migration analysis, migration speed, total path length and net displacement were calculated from centroid coordinates, and directionality was calculated as net displacement divided by total path length. For bacterial-association analysis, cells were classified as GFP-PA positive when GFP-PA signal was detected within the cell mask or within an adjacent 2-μm-wide pericellular region, and as GFP-PA containing when GFP-PA signal was detected within the cell mask.

### Statistics and reproducibility

Statistical analyses were performed using Origin (OriginLab). Measurements were obtained from multiple fields of view and individual cells across independent mice. For time-lapse analyses, repeated measurements from the same tracked cell were used to calculate temporal variability or migration parameters for each cell. Comparisons between two groups were performed using unpaired two-sided Student’s t-tests. Comparisons among three groups were performed using one-way ANOVA followed by Tukey’s multiple-comparisons test. The statistical test used for each comparison and the numbers of mice, fields of view, and cells are provided in the corresponding figure legends. In addition, FOV- or cell-level measurements were aggregated within each mouse and analyzed using the individual mouse as the independent unit. Mouse-level analyses showed effects in the same direction as the primary FOV- or cell-level analyses. P < 0.05 was considered statistically significant.

## Supplementary Material

Supplementary video legends

**Supplementary video 1 Dynamic Ca**^**2+**^ **activity in PMNs moving through the pulmonary capillary network.** Representative ratiometric intravital Ca^2+^ imaging of fields of view from a Vehicle-treated mouse (left) and a GsMTx4-treated mouse (right). White indicates pulmonary vascular structures. Scale bar, 50 μm. Time, min:s.

**Supplementary video 2 Ca**^**2+**^ **elevation in PMNs accompanies cell deformation during pulmonary capillary passage.** Representative enlarged single-cell ratiometric intravital Ca^2+^ imaging of PMNs moving through pulmonary microvessels in a Vehicle-treated mouse (top) and a GsMTx4-treated mouse (bottom). Intracellular Ca^2+^ signal increases as cells undergo marked deformation while passing through narrow capillary segments. White indicates pulmonary vascular structures. Scale bar, 10 μm. Time, min:s.

**Supplementary video 3 PMN**_**cyt**_**s appear in the inflamed lung.** Representative two-photon intravital imaging of PMNs (red), nuclei (green) and pulmonary microvessels (blue) 3 h after intraperitoneal LPS administration. Anuclear PMN-derived cytoplasts (PMN_cyt_s) are observed within the pulmonary microvasculature under endotoxemic conditions. White arrows indicate nucleated PMNs, and red arrows indicate anuclear PMNs. Scale bar, 50 μm. Time, min:s.

**Supplementary video 4 PMN**_**cyt**_**s show greater motility than nucleated PMNs.** Representative enlarged two-photon intravital imaging of nucleated PMNs and PMN_cyt_s 3 h after intraperitoneal LPS administration. In the inflamed lung, PMN_cyt_s show greater motility than nucleated PMNs. Red, PMNs; green, nuclei; blue, pulmonary microvessels. Scale bar, 10 μm. Time, min:s.

**Supplementary video 5 PMN**_**cyt**_**s show greater temporal morphological plasticity than nucleated PMNs.** Representative enlarged two-photon intravital imaging of nucleated PMNs and PMN_cyt_s 3 h after intraperitoneal LPS administration. PMN_cyt_s show more pronounced shape changes than nucleated PMNs. Red, PMNs; green, nuclei. Scale bar, 10 μm. Time, min:s.

Supplementary Files

This is a list of supplementary files associated with this preprint. Click to download.

• Supplementaryvideo1.mp4

• Supplementaryvideo2.mp4

• Supplementaryvideo3.mp4

• Supplementaryvideo4.mp4

• Supplementaryvideo5.mp4

## Data Availability

Source data underlying Figs. 1–5 are provided with this paper. Raw time-lapse microscopy datasets supporting the findings of this study are available from the corresponding author upon reasonable request owing to their large file size.

## Code availability

The original CoVSTii image-stabilization software and source code are publicly available as described in our previous publication^3^. The updated version used in the present study is available from the corresponding author upon reasonable request.
